# A Mobile Phone–Based App for Use During Cognitive Behavioral Therapy for Adolescents With Anxiety (MindClimb): User-Centered Design and Usability Study

**DOI:** 10.2196/18439

**Published:** 2020-12-08

**Authors:** Amanda Newton, Alexa Bagnell, Rhonda Rosychuk, Janelle Duguay, Lori Wozney, Anna Huguet, Joanna Henderson, Janet Curran

**Affiliations:** 1 Department of Pediatrics University of Alberta Edmonton, AB Canada; 2 IWK Health Centre Halifax, NS Canada; 3 Department of Psychiatry Dalhousie University Halifax, NS Canada; 4 Centre for Addiction and Mental Health University of Toronto Toronto, ON Canada; 5 School of Nursing Dalhousie University Halifax, NS Canada

**Keywords:** anxiety disorders, mobile apps, adolescents, usability testing, development, design, anxiety

## Abstract

**Background:**

Mobile device–based tools to help adolescents practice skills outside of cognitive behavioral therapy (CBT) sessions for treating an anxiety disorder may lead to greater treatment gains.

**Objective:**

This study aimed to develop, design, and test the acceptability, learnability, heuristics, and usability of MindClimb, a smartphone-based app for adolescents with anxiety to use between CBT sessions to plan and complete exposure activities using skills (cognitive, relaxation, exposure practice, and reward) learned in treatment.

**Methods:**

This 3-phase study took place from August 2015 to December 2018. In phase 1, the app was designed and developed in consultation with young people and CBT therapists to identify desired functions and content. Feedback was subjected to thematic analysis using a general inductive approach. In phase 2, we conducted 2 high-fidelity testing sessions using the think-aloud approach (acceptability, learnability, usability) and 10-item System Usability Scale with 10 adolescents receiving CBT. The high-fidelity MindClimb app was evaluated by 5 app developers based on Nielsen’s usability heuristics and 5-point severity ranking scale. In phase 3, a total of 8 adolescents and 3 therapists assessed the usability of MindClimb during CBT sessions by recording the frequency of skills practice, use of MindClimb features, satisfaction with the app, and barriers and facilitators to app use during treatment.

**Results:**

Feedback from phase 1 consultations indicated that the app should (1) be responsive to user needs and preferences, (2) be easy to use and navigate, (3) have relevant content to the practice of CBT for anxiety, and (4) be aesthetically appealing. Using this feedback as a guide, a fully functional app prototype for usability testing and heuristic evaluation was developed. In phase 2, think-aloud and usability data resulted in minor revisions to the app, including refinement of exposure activities. The average system usability score was 77 in both testing cycles, indicating acceptable usability. The heuristic evaluation by app developers identified only minor errors (eg, loading speed of app content, with a score of 1 on the severity ranking scale). In phase 3, adolescents considered app features for completing exposure (6.2/10) and relaxation (6.4/10) modestly helpful. Both adolescents (average score 11.3/15, SD 1.6) and therapists (average score 10.0/12, 2.6 SD) reported being satisfied with the app.

**Conclusions:**

The user-centered approach to developing and testing MindClimb resulted in a mobile health app that can be used by adolescents during CBT for anxiety. Evaluation of the use of this app in a clinical practice setting demonstrated that adolescents and therapists generally felt it was helpful for CBT practice outside of therapy sessions. Implementation studies with larger youth samples are necessary to evaluate how to optimize the use of technology in clinical care and examine the impact of the app plus CBT on clinical care processes and patient outcomes.

## Introduction

In recent years, mobile device–based apps have been regarded as an opportunistic and potentially effective adjunct to cognitive behavioral therapy (CBT) for adolescent anxiety disorders. In the treatment context, an app is considered an ecological momentary intervention (EMI) [1] for in vivo skills coaching in an adolescent’s natural settings (ie, at home, school, etc instead of a therapist’s office) when it is most needed during daily life (ie, in real time). Conventionally, skills practice between treatment sessions has been supported through paper-based workbook activities and assignments. However, this approach to skills practice can be limited if the workbook and assignments are not an integral or natural part of an adolescent’s daily life, unlike the use of mobile devices, such as smartphones.

Adolescents can realize greater treatment gains when they practice CBT skills outside of formal treatment (eg, at home or school) [2-4], yet adherence to and persistence with skills practice are common challenges for adolescents during CBT. Skills take a taxing effort, as purposely and repeatedly being in feared situations is mentally and physically uncomfortable. The use of an EMI app during CBT offers several solutions to these challenges. An app can provide structure to skills practice to promote consistency between skills practiced in a treatment session and those practiced in real-life practice situations, which can be less predictable than treatment contexts. Push notifications from smartphone calendars can remind adolescents to practice skills and engage in relaxation and self-rewarding, pleasurable activities to offset discomfort, encourage self-care, and reinforce hard work.

While recent systematic reviews of mobile health (mHealth) apps have identified several CBT-based apps that adolescents with anxiety can use independently to practice exposure, relaxation, and coping strategies [5,6], one app, SmartCAT, has been specifically designed as an EMI app for patient and therapist use during CBT [7]. The SmartCAT app prompts patients to use CBT skills learned in treatment and, via the web, allows therapists to monitor and reward skill use and communicate with patients using an integrated clinician portal for secure 2-way communication. In a feasibility study of SmartCAT, 9 children and adolescents aged 9 to 14 years rated the app as easy to use and used it regularly during treatment [7]. In a follow-up open trial with a larger sample of same-aged patients (n=34), users were highly satisfied with the app and used it an average of 12 times between each in-person CBT session. Other findings included improvements in CBT skills targeted by the app [8].

Our team was interested in developing an EMI app for adolescent (aged 13-18 years) use that would interface with native smartphone features, information, and hardware. As a native app, it would be installed directly from an app store (eg, Google Play or Apple’s App Store) onto the smartphone and would not require access to Wi-Fi or a data plan. A native app would also allow our team to take advantage of cross-functional device abilities (eg, push notifications, interaction with calendar) [9,10] and multimedia opportunities (eg, use of camera, voice recordings, videos) to personalize planning and skills practice. We believed that adolescent use of a personal smartphone for exposure activities, relaxation strategies, and self-reward would help promote independent skills practice in real time and provide adolescents with a sense of ownership of their treatment goals, since they would be integrated into their personal device. We also wanted the app to be simple in design, as an app that is not easy to use during stressful skills practice would likely not be used by adolescents. The app design process incorporated academia, end users, and industry to create an engaging and useful intervention tool [11]. The objectives of this study were to develop, design, and test the acceptability, learnability, heuristics, and usability of this native app, which we called MindClimb.

## Methods

### Study Overview

**Figure 1:**
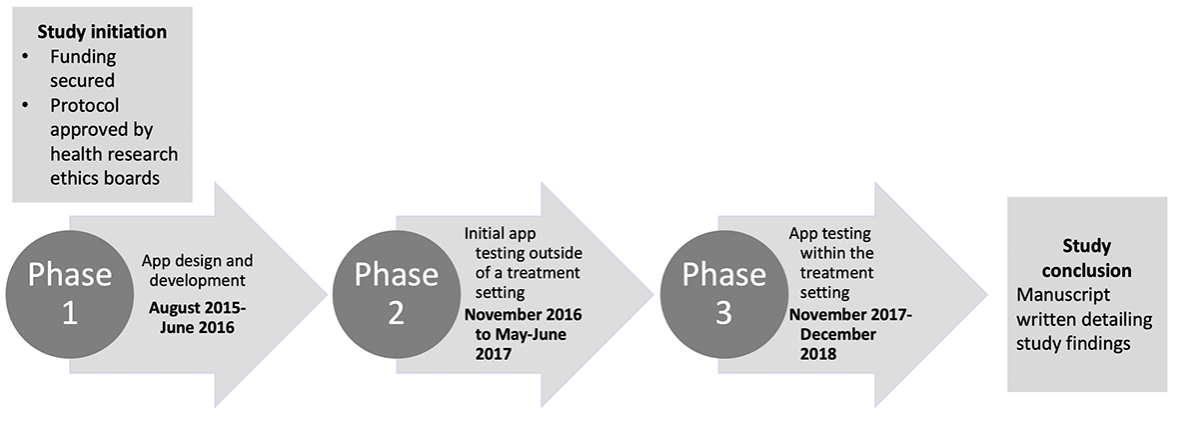
Study timeline.

The study took place from August 2015 to December 2018 (Figure 1) and was conducted in 3 phases. We worked with an app designer and developer to create the app using a user-centered design approach during each study phase [12]. The study design is consistent with recommendations on mHealth technology development and evaluation, including user experience design, development, and alpha and beta testing [13]. Informed assent or consent was obtained from all participants prior to participation. In phase 1, the MindClimb app was designed and developed in consultation with youth and CBT-trained clinicians. In phase 2, adolescents in treatment for an anxiety disorder evaluated the app for acceptability, learnability, and usability in 2 testing cycles that took place outside of the formal treatment setting. In this phase, we also asked app developers to evaluate the app using a set of rules (heuristics) to measure the usability of the user interface. For this phase, we used Nielsen’s heuristics [14]. In phase 3, app usability was assessed by adolescents and therapists during CBT treatment for anxiety. This involved reviewing the frequency of app use for skills practice between CBT sessions, satisfaction with app features, and barriers and facilitators to app use. The study was led out of the University of Alberta (Edmonton, Alberta, Canada) and Dalhousie University (Halifax, Nova Scotia, Canada). The University of Alberta and the IWK Health Centre’s Research Ethics Boards approved the study.

### Participants

We recruited a convenience sample of adolescents, young adults, and CBT therapists to provide initial, varied perspectives on app design. Our goal was to recruit 7 to 10 participants. We invited individuals aged 12 to 24 years from across Canada who were members of the Centre for Addiction and Mental Health’s National Youth Advisory Committee (NYAC) and CBT therapists specialized in outpatient anxiety treatment at the IWK Health Centre in Halifax, Nova Scotia, Canada, to participate. CBT therapists from the same setting were also invited to participate in phase 3 if one of their patients was recruited into this study phase.

For all phases, we recruited purposeful samples of adolescents aged 13 to 18 years from an outpatient program at the IWK Health Centre in Halifax, Nova Scotia, Canada. At the IWK Health Centre, adolescents are treated until their 19th birthday. Adolescents receiving outpatient treatment at this setting scored ≥25 on the Screen for Child Anxiety Related Emotional Disorders (SCARED) [15], indicating a clinical level of anxiety symptoms. To participate, adolescents needed to be fluent in English. As sex differences are noted in mobile phone use [16], we hoped both adolescent boys and girls would participate. Youth identifying with any gender were welcome to participate, as we were not testing sex- or gender-based differences in app use in this study. Honorariums were provided to participants (phase 1 and 2: Can $25 gift card [US $19.14]; phase 3: Can $40 gift card [US $30.61]). Consistent with recommendations in the literature, our recruitment goals for phases 1 and 2 were 7 to 10 adolescents (phase 1) and 10 adolescents (phase 2) [14,17]. In phase 3, we aimed to enroll 21 adolescents and 7 CBT therapists (with each therapist treating 3 of the enrolled adolescents) to comprise a total sample size of 28 participants. This sample size was chosen assuming a 30% dropout rate for clinicians and patients (5 clinicians with 3 patients per clinician, for a total of 15 participants), which would still allow detection of an effect size of at least 1.1 in the mean change in frequency of app use (between last and first weeks of use; 2-sided, 1-sample *t* test, 80% power, type I error rate of 0.05). PASS (NCSS Statistical Software) was used to determine the detectable effect size, and the sample size was adjusted for potential intracluster correlation (clinicians were considered a cluster; ρ=0.1) using an inflation factor.

We also recruited a convenience sample of app developers in phase 2 via email using professional contacts identified by our app developer. To be eligible to participate in the heuristic evaluation, developers needed to have postsecondary training in human-computer interaction and experience in designing and evaluating health apps for mobile devices. We considered 5 developer participants to be an adequate sample size for the heuristic-based evaluation [14,17].

### Procedures

#### Phase 1

**Figure 2:**
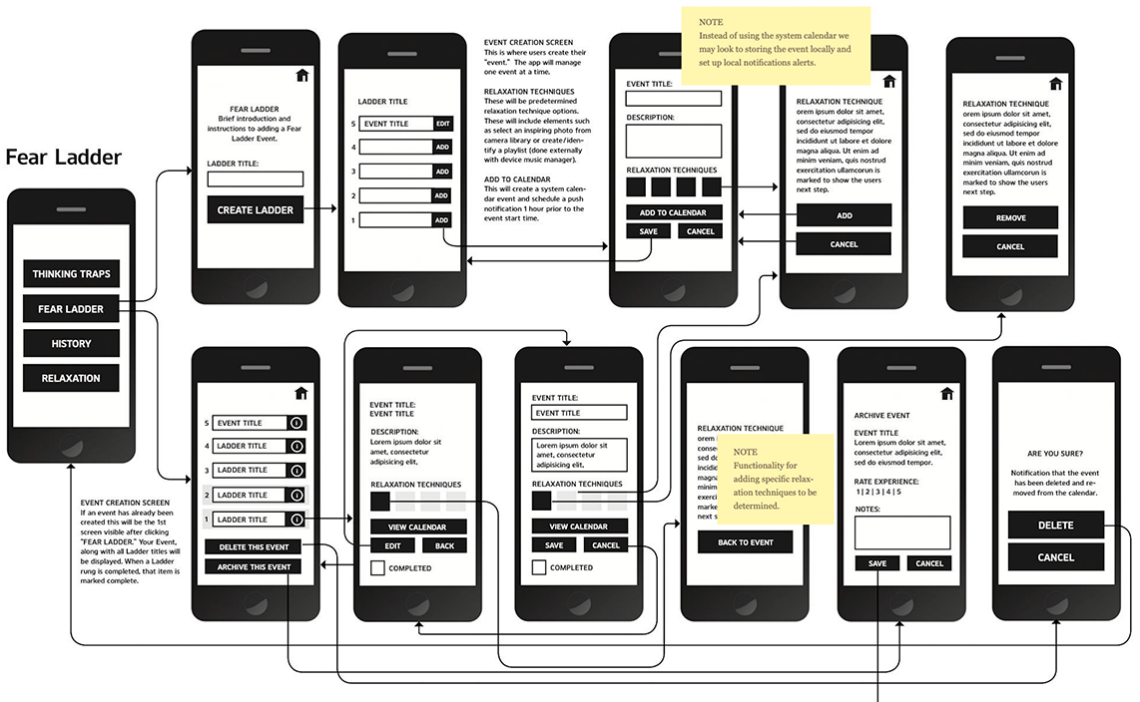
Screenshot of an initial MindClimb wireframe.

App development began with wireframing, a process in which our team created black-and-white pictures of MindClimb app screens and arranged them to determine how users would navigate and use the app (Figure 2). We organized the MindClimb wireframes around guiding adolescent users through the development of 3 main components: (1) fear ladders, (2) relaxation, and (3) thinking traps.

First, fear ladders are a tool designed to help adolescents work on their exposure goals. Our goal was to allow adolescents to create, select, and manage multiple ladders. Events for each ladder would be synced with the phone’s calendar app, and phone notifications would be used to remind and cue adolescents of upcoming events. After completion of the exposure, adolescents could rate the activity in terms of its difficulty. Ladders could also be archived (“history” tab on the wireframe).

Second, the relaxation section would offer information about relaxation techniques that could be used as strategies before and after exposure events and for general self-care.

Third, the thinking traps section would contain information on and examples of common cognitive errors that could occur during planned exposure activities.

All user actions were mapped out and the details of each interaction were defined. Content (text) was later created to support app functions and tasks.

**Figure 3:**
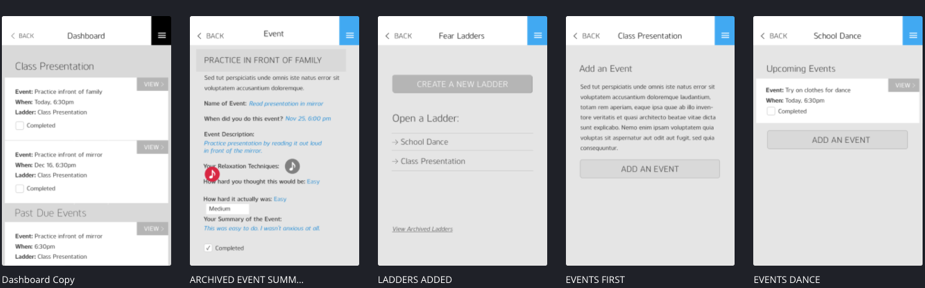
A screenshot of several phone screens for the predesign prototype of the MindClimb app.

We used the wireframes to create a predesign prototype of the MindClimb app using InVision (InVisionApp Inc), a digital product design platform [18] used by our app developer. The predesign prototype was visually similar to the black-and-white line art used in the wireframes (Figure 3).

The predesign prototype was shared with NYAC members and CBT clinicians during separate 1-hour brainstorming sessions. The youth session was held via a web conference and led by 2 NYAC youth engagement facilitators who were collaborators on the study [19]. Youth were prompted to generate ideas on app aesthetics (ie, look and feel) and desired features of a user experience (eg, what app features would ease use) (see Multimedia Appendix 1). The clinician session was held in person at the IWK Health Centre and facilitated by a research team lead and coordinator and the app developer. As therapists were oriented to the predesign prototype, they were prompted to discuss the flow and approach of the app’s features and whether any essential treatment components were missing from the app (section 1 in Multimedia Appendix 1). We summarized the ideas generated from both sessions by themes and incorporated them into the development and design plans for the app.

We initiated app design after the predesign consultations concluded. During app design, our team completed 3 steps: (1) review of the style board, a collage of different design styles and elements, to establish the design direction of the app, (2) approval of a creative brief to establish the aesthetic approach to be used for the app, and (3) approval of the interface design by reviewing several visual mock-ups of wireframe screens that were based on the direction defined in the creative brief. The result of these steps was a design plan that was used by our app developer to create a low-fidelity MindClimb prototype in the InVision platform [18].

The low-fidelity prototype underwent evaluation by adolescents receiving outpatient CBT treatment. Adolescents participated in a digitally recorded focus group facilitated by a research team lead and coordinator and the app developer. As adolescents interacted with the prototype, they were prompted to discuss app features and use, including difficulty in creating exposure events and navigating the app (verbal ratings on a scale of 1 to 5: 5=hard, 1=easy) (section 2 of Multimedia Appendix 1). We transcribed the recorded sessions and thematically analyzed the discussions using a general inductive approach [20]. We used the themes and verbal ratings to finalize the programming on an iOS version of the MindClimb app that was fully functional (a high-fidelity prototype).

#### Phase 2

We used a mixed-method approach in 2 iterative testing cycles to refine the high-fidelity MindClimb prototype. In both cycles, we asked adolescents to evaluate the usability and acceptability of the prototype, and in cycle 2 we also evaluated its learnability. In cycle 2 we asked app developers to conduct a heuristic evaluation of the prototype to identify usability problems with the user interface design [14,21]. During testing, MindClimb was supported by TestFlight (Apple Inc) [22], an app designed to support beta testing that was used by our app developer.

Adolescent testing sessions took 1 hour to complete and were led by a research team lead and coordinator and the app developer. The sessions were structured according to a think-aloud testing guide (Multimedia Appendix 2) and digitally recorded to facilitate data analysis. Adolescents who did not own or have access to an iOS operating system smartphone that was required to download MindClimb were provided with a smartphone or tablet on which to use MindClimb during the testing cycle.

In cycle 1, adolescents first participated in a walk-through exercise on how to create an exposure activity (fear hierarchy) using the app’s ladder function. Adolescents were then asked to complete their own ladder, and facilitators engaged them during this task by posing questions from the testing guide to document experiences with task completion. Field notes documented by the facilitators were used to inform results interpretation. Adolescents were encouraged to use the app on their own before cycle 2 testing (one week after the first cycle). We chose this timeframe because we wanted to understand learnability in the timeframe in which adolescents would use the app during CBT (ie, between weekly therapy sessions). Cycle 2 proceeded similarly to cycle 1 and included gathering feedback regarding adolescents’ use of the app during the previous week. At the end of both cycles, adolescents rated their experience with the app by completing the 10-item System Usability Scale (SUS) [14].

Data from the think-aloud activity were examined using a template approach to analysis [23]. We reviewed the findings from cycle 1 after the session to allow the developer to make necessary modifications to MindClimb prior to cycle 2. We interpreted mean SUS scores using published recommendations: a mean score >70 indicated acceptable usability, 50 to 70 indicated marginal usability, and <50 indicated that the app was not acceptable (score range was 0-100). Given the user-centered process that we undertook for app development, we hypothesized that adolescents would report high acceptability and learnability, as supported by the qualitative think-aloud data and an acceptable usability score (≥70).

Following adolescent testing, we asked mobile app developers to evaluate MindClimb based on Nielsen’s usability heuristics using a 5-point severity ranking scale (SRS) [17]. Median scores from the SRS were interpreted as follows: 0=none, 1=cosmetic, 2=minor, 3=major, and 4=catastrophic [24]. We hoped that developers would report a low level of errors (ratings of 1 or 0) using the SRS. Any median SRS scores ≥1 were addressed in a final iOS version of the app, and an Android version was then built.

#### Phase 3

In the final phase, we conducted a case series study to assess the usability of MindClimb among adolescents and therapists during group CBT for anxiety. The group therapy setting was a more convenient approach to recruit from and to study app use during real-world clinical care. Specific usability objectives were to (1) track frequency of skills practice between CBT sessions through self-report of ladder use (use of exposure hierarchies) and other MindClimb features as part of CBT and (2) identify satisfaction as well as barriers and facilitators to MindClimb use during CBT.

Following enrollment, a research coordinator collected adolescent demographic data (age and gender) and contact information (email address and phone number) and recorded the adolescent’s preference for future contact (phone, text, or email). Therapists were asked to provide their names and contact information (phone and email).

A research team lead, research coordinator, and app developer provided training sessions for adolescents and clinicians on downloading MindClimb to their smartphones and using the app in conjunction with weekly CBT sessions; therapists were not required to download the app, as they would not be actively using it but rather, supporting their patient during use. MindClimb was available for download in both the iOS App Store and Google Play Store. Training was guided by a review of a MindClimb quick start user guide provided to participants. During training, therapists could also download the app for the purposes of understanding the app’s functions so that discussion of app use could be incorporated into treatment sessions, and they were provided the same review of the MindClimb quick start user guide.

**Figure 4:**
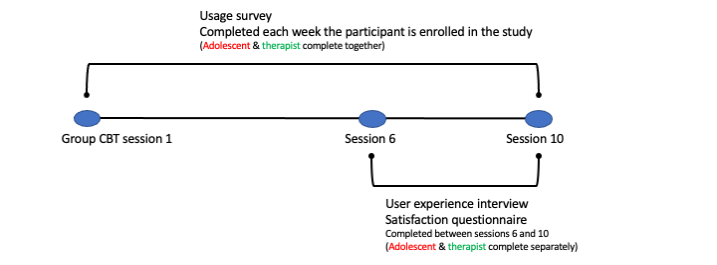
Phase 3 data collection timeline. CBT: cognitive behavioral therapy.

Data were collected throughout the group therapy period (Figure 4). Adolescents began using the app with sessions 3 to 9 to coincide with treatment content on exposure activities. The use of key CBT features in MindClimb—ladders (exposure hierarchies), thinking traps, and relaxation strategies—was assessed weekly, with therapists completing the survey with each adolescent to report how frequently the adolescent used MindClimb between treatment sessions and rating the helpfulness of features they used over the same period on a scale of 1 (least helpful) to 10 (most helpful) (Multimedia Appendix 3). After 6 to 7 treatment sessions, adolescents and therapists were contacted to report satisfaction and experience with using MindClimb during treatment. Satisfaction with app use was measured using a study-created questionnaire based on the Client Satisfaction Questionnaire [25] for adolescents (5 questions asked; score range of 0-15, with higher scores indicating higher satisfaction) and therapists (4 questions asked; score range of 0-12, with higher scores indicating higher satisfaction) (Multimedia Appendix 4). User experience interviews were conducted by a research team lead (clinician interviews) or research coordinator (adolescent interviews). Therapists completed the interview in person; adolescents had the option to complete the interview over the phone or through online chat (eg, using iMessage). If an adolescent was not comfortable completing the interview over the phone or using online chat, they had the option to complete an online version of the interview in SurveyMonkey that used open fields for text entry. None of the adolescents in phase 3 chose this online option. Interviews were based on a semistructured guide featuring the 14 domains of the Theoretical Domains Framework (TDF) [26], with versions for therapists (Multimedia Appendix 5) and adolescents (Multimedia Appendix 6). The TDF provided a useful approach to identifying experiences, barriers, and facilitators to MindClimb use. Interviews were digitally recorded and transcribed.

We used descriptive statistics (eg, means with standard deviations, proportions and percentages, medians with range) to summarize demography and MindClimb satisfaction and use. We also used box plots to visually display MindClimb use. We used directed content analysis to synthesize interview data [27]. We were unable to recruit our intended sample size of therapists and patients, so intracluster correlation was not determined for continuous outcomes, and adjusted mean change scores were not calculated.

## Results

### Phase 1

Predesign consultations on MindClimb were held in November 2015 with 6 NYAC youths via a web conference and in December 2015 with 3 therapists (9 participants in total). Feedback provided on the predesign prototype is summarized in Table 1, alongside the impact of this feedback on MindClimb development and design. Feedback centered on 4 areas for app development and design: (1) the app should be responsive to user needs and preferences, (2) the app should contain features relevant to the practice of CBT for anxiety, (3) the app should be easy to use and navigate, and (4) app design should prioritize aesthetics.

**Table 1:** Summary of the feedback provided during the predesign consultations.

Development area and feedback		Impact on MindClimb development and design
**The app should be responsive to user needs and preferences**		
	Ensure app connectivity with phone calendar (youth)	No change made, as this feature was already intended.
	Do not require Wi-Fi connection (youth)	No change made, as this feature was already intended.
	Personalization should be possible (youth)	Users will have the ability personalize exposure activities and coping strategies.
	Make the app interactive (youth)	The app will contain interactive features, such as rewards and points for app use and pop-ups with positive affirmations.
	Build in rewards; completion of exposure activities unlocks new reward content (therapist)	Completion of exposure activities will unlock new reward content.
**The app should contain features relevant to the practice of CBT^a^ for anxiety**		
	More information on self-care strategies (therapist)	The app will contain text and videos on self-care strategies.
	Consider a live chat with counselors (youth)	No change made, as it is beyond the scope of the purpose of the app.
	Include a “tools” section that contains static information along with interactive components (therapist)	The app will contain text and videos on self-care strategies. Interactivity will be limited to videos.
	Include an “other” option under suggested self-care and coping strategies to allow patients to identify a personal strategy not listed	The app will include an “other” option under suggested self-care and coping strategies.
	Ensure users can repeat scheduled exposure events (therapist)	Users will have the ability to retry and reschedule events.
	The app dashboard should list overdue events before upcoming events (therapist)	Events created in the app will appear in a single stream, with overdue events flagged as being overdue. Users will have the option to view and reschedule these events from the dashboard.
**The app should be easy to navigate**		
	Ensure the app is not cluttered and has clean lines (youth)	Content for each app section will be brief. Visual clutter will be avoided.
	Make sure there is less content on screens (ie, more pages or screens for content) (youth)	Each screen will support a single action for the user.
	Tabs to facilitate use (youth)	No change made; the main dashboard will be used.
	Images for guiding steps or how to use (youth)	Videos will be used to provide information on how to practice specific relaxation techniques; images will not be used for guiding the creation of exposure activities, but sample fear hierarchies (ladders) will be provided.
	Ensure that it is easy to move between functions (youth)	App functions will be sequential and logical.
	Self-explanatory icons (youth)	Each app icon will reflect the section content that it represents.
	Ensure that it is easy to scroll through content (youth)	Content for each app section will be brief so that scrolling down for information or functions will not occur.
	Ensure transitions between functions are quick (no lagging) (youth)	Push notifications will be limited to reduce the likelihood of noticeable lag in the running of the app.
**App design should prioritize aesthetics**		
	Big lettering for titles, large fonts for important content (youth)	All feedback incorporated into the creative direction and design options (storyboard) developed for the app.
	No advertisements (youth)	Incorporated into design options.
	“Calm” aesthetic (youth)	Incorporated into design options.
	Bright colors, neutral colors, not too many colors (youth)	Incorporated into design options.
	Twitter-style text, brief and concise (youth)	Incorporated into design options.
	Have a good graphic artist/designer (youth)	Incorporated into design options.
	Use sans serif font (youth)	Incorporated into design options.

^a^CBT: cognitive behavioral therapy.

**Figure 5:**
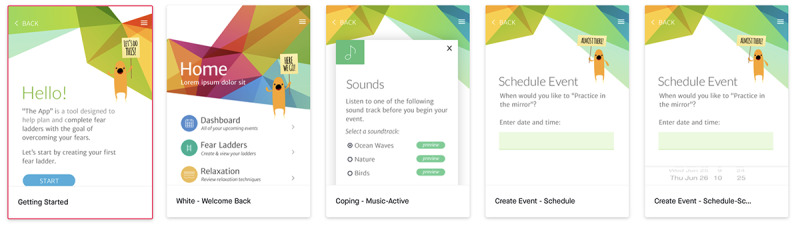
Screens from the low-fidelity MindClimb prototype.

The app was further developed based on youth and clinician feedback. A low-fidelity MindClimb prototype was then created. Screens from the prototype are presented in Figure 5. The prototype native app had full functionality and could be used by youth and therapists from an iOS device without requiring an internet connection.

Feedback on the low-fidelity prototype was collected in June 2016 from 4 adolescents receiving outpatient CBT. We were unable to recruit our intended minimum of 7 participants. Participating adolescents reported preferring a colorful palette with white accents. They understood what the MindClimb app was for by looking at it and found it easy to create exposure events in the app (4 ratings of “1”). Ease of navigation was also rated as easy (ratings of “1” and “2”) and app sections were not confusing. Adolescents indicated that preferred terms in the app were “step ladders” rather than “fear ladders” for exposure activities and “helpful thoughts” versus “thinking traps” in order to shift to more positive or neutral wording. The app was modified to use “step ladders,” but we chose not to use “helpful thoughts,” as this change in wording did not accurately convey the content in the thinking traps section of the app. However, we did review the relaxation section of the app to ensure helpful thoughts were conveyed.

### Phase 2

Testing the high-fidelity MindClimb prototype took place in November 2016. A total of 10 adolescents participated in cycle 1, and 7 of these adolescents also participated in cycle 2. Of the 10 participants, 5 were female (50%) and 3 were male (30%); gender was not documented for 2 adolescents. The mean age across 7 adolescents was 15.1 (SD 0.90) years; age was not documented for 3 adolescents. The mean SUS score from both cycles was 77, indicating acceptable usability. Results from the think-aloud activities revealed only minor issues with the app. Issues identified by the adolescents in the testing cycles and changes made to the app are outlined in Table 2. Changes to the app were made prior to the heuristic-based evaluation.

**Table 2:** Summary of adolescent feedback generated during usability testing cycles that was incorporated into MindClimb.

App feature and feedback		Changes applied
**Security and privacy**		
	Cycle 1: password protection is important	Incorporated an option for the user to create a PIN^a^ that must be entered each time the app is opened
	Cycle 2: “a password to get into the app or ladders”	Syncing of events with main calendar is optional
**Notifications**		
	Cycle 1 generated varied feedback on the frequency of user notifications, including wanting daily reminders, reminders 1 to 2 times per week, and notifications after a period of 1 to 2 weeks of inactivity	Added single “We’ve missed you!” notification to pop up after 1 week of having not used the app
	Cycle 2: alerts were described as sometimes overwhelming and stressful. Adolescents wished for an option to turn off notifications	Users will receive 1 notification alert when their event is scheduled to occur
**Step ladder creation and management**		
	Cycles 1 and 2: a scale of 1 to 10 to rate the difficulty of exposure activities is more appropriate than 1 to 5	Difficulty scale for exposure activities changed to 1 to 10
	Cycle 2: adolescents wanted the option to complete an exposure activity several times before proceeding to the next step in their ladder	Added the ability to repeat an event
	Cycle 2: scheduling specific dates and times for each exposure activity might be difficult to plan; app should allow users to “opt out of dates” and dates should not be mandatory	Selection of dates for exposure activities is optional
	Cycle 2: adolescents wanted ability to change the order of exposure activities and an easy way to “move the steps around”	Exposure activities, regardless of difficulty rating, can be rearranged by dragging and dropping
	Cycle 2: adolescents wanted the ability to not have a coping strategy attached to an exposure activity	Activities can be created without selecting a coping strategy
**Positive reinforcement and rewards**		
	Cycle 1: “Quotes would be good. Ones that make you smile more or positivity”; creatures were a popular reward, especially the ability to upgrade characters; “increase the content” that the monster character says	Incorporated random short positive messages provided by the monster characters at various points within app
	Cycle 2: adolescents wanted monster characters to relate quotes to them; “it’s fun to use, but the point system doesn’t do anything right now. If the points system unlocked another guy (character)”	Added 3 additional monster characters (total of 4) that can be unlocked for completing activities within the app
**Positive self-talk**		
	Cycle 2: adolescents wanted to be able to record “own memo (voice), positive self-talk”	Allow users to record their own positive self-talk messages and replay them during exposure activities
**Relaxation techniques and thinking traps**		
	Cycle 1: adolescents wanted a voice that could walk them through the relaxation techniques	Added voice narration and video as a way for users to access selected relaxation techniques during events
	Cycles 1 and 2: too much text on deep breathing and thinking traps	Text edited for length and clarity
	Cycle 2: techniques learned in CBT^b^ are not in the app	Added “acting as if” relaxation techniques

^a^PIN: personal identification number.

^b^CBT: cognitive behavioral therapy.

The heuristic evaluation took place between May and June 2017. A total of 5 developers, all male, participated. For the majority of the app’s usability heuristics, the median score indicated no issue. A cosmetic issue regarding load time was identified for the error prevention heuristic and was corrected. Results from the evaluation are presented in Table 3.

**Table 3:** Severity ranking scale results from mobile app developers.

Usability heuristic	Median score^a^	Developer comments
1. Visibility of system status	0	“It would be nice to have to see your points or progress somewhere all the time” [Developer 5]
2. Match between system and the real world	0	“Instructions on ‘Create an Event’ page not very descriptive. Could include example of an event. (e.g., Practice in front of mirror)” [Developer 1]
3. User control and freedom	1	“No immediate ‘undo’ option for event completion choices, but events can be edited anytime to change completion choice” [Developer 2]
4. Consistency and standards	1	“If different terminology is used in treatment it may take a moment for the terms used in MindClimb to register with users” [Developer 3]
5. Error prevention	0	“The app sometimes takes a long time (~20secs) to move from the splash screen to the home screen on iPhone 5S” [Developer 2]
6. Recognition rather than recall	0	“When adding a relaxation technique, I selected a category and chose a specific technique. When you tap Save and return to the category selection screen, it would be nice to see the specific technique you chose and not only the category selected before hitting next” [Developer 5]
7. Flexibility and efficiency of use	0	“Not a lot of system tailoring present, but given the target audience and system scope, likely not required or useful” [Developer 2]
8. Aesthetic and minimalist design	0	“Some subheading may not be needed (e.g., ‘select a ladder to view its events’)” [Developer 1]
9. Help users recognize, diagnose, and recover from errors	0	“Did not encounter any error conditions” [Developer 5]
10. Help and documentation	0	“Help documentation was readily available on all screens” [Developer 5]

^a^Heuristic scoring range: 0=none, 1=cosmetic, 2=minor, 3=major, and 4=catastrophic.

**Figure 6:**
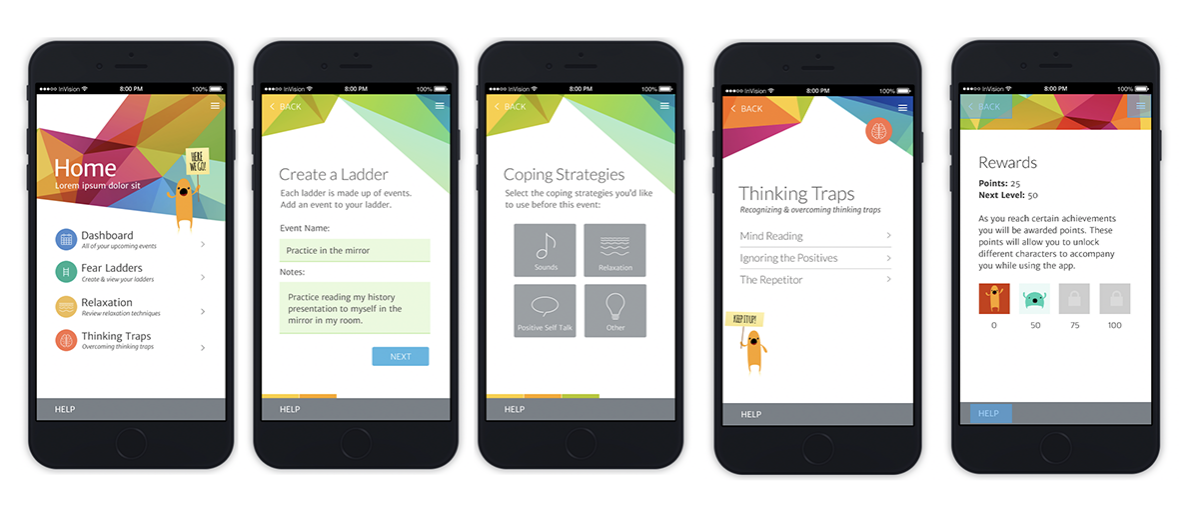
Screenshots of key content domains for MindClimb.

Screenshots of the final version of MindClimb are provided in Figure 6. Based on the high-fidelity evaluations from adolescents and app developers, the final version of the app has the following key content and functions: (1) step ladders, (2) dashboard, (3) relaxation, (4) thinking traps, (5) rewards, (6) settings, and (7) help sessions.

First, the app contains step ladders, formerly termed fear ladders. Up to four coping strategies built into the app (eg, audio clips of soothing sounds, text instruction and videos of relaxation techniques) can be identified for each event. When creating ladders, the adolescent indicates how difficult they think it will be to complete the exposure (scale of 1 [easy] to 10 [hard]); after completion of the exposure, adolescents can rate the activity again in terms of its difficulty to actually carry out. Users are able to repeat exposures as part of the step ladder features; ladders can be archived for review of progress and activities.

Second, the dashboard contains all of the upcoming events created for each step ladder. The dashboard provides a quick overview of scheduled events sorted by date.

Third, for the relaxation feature, text and audiovisual clips are available in conjunction with information about relaxation techniques that can be used as strategies before and after exposure events and for general self-care.

Fourth, the thinking traps section contains brief information on and examples of common cognitive errors.

Fifth, a rewards section tracks adolescent progress with step ladder events. Points are awarded for event and ladder completion; different point levels allow the adolescent to unlock different MindClimb characters that appear with encouraging messages while the app is being used.

Sixth, a settings screen allows the adolescent to protect MindClimb with a 4-digit personal identification number (PIN), set a security question and answer should the PIN be forgotten, and turn on calendar syncing so that MindClimb events sync with the phone’s calendar app.

Last, a help session supports use in each content domain (eg, sample ladders are provided in the step ladders section).

### Phase 3

A total of 8 adolescents (all girls) and 3 therapists (all women) used MindClimb during group therapy between November 2017 and December 2018. Another 3 adolescents consented to participate, but 1 withdrew from the study and 2 did not download the app for study participation. The 2 adolescents who did not download the app for use did not provide information on why they chose not to use the app. The mean age across the participating adolescents was 14.0 (SD 1.5) years. The median SCARED score reported by adolescents was 56 (range of 46-62); the median SCARED score reported by parents regarding their children was 46 (range of 23-60).

**Figure 7:**
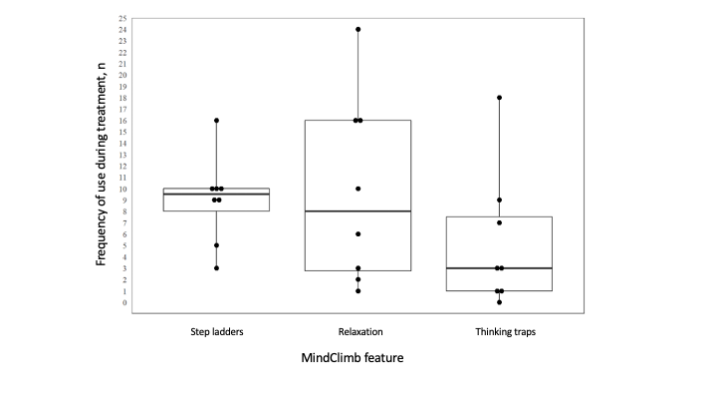
Median use of MindClimb features over 7 sessions of group cognitive behavioral therapy.

Over the group treatment sessions, MindClimb use began during session 3 (with 2 adolescents), session 4 (with 1 adolescent), session 5 (with 3 adolescents), or session 6 (with 2 adolescents). All the adolescents reported using the app between sessions, and use ranged between features and adolescents (Figure 7); none of the adolescents reported use in the final week of treatment. The largest range of use occurred with the relaxation feature of the app, with some users using it infrequently (once) and some using it regularly (24 uses over a 4-week period). Use of the thinking traps feature was also varied (range of 0-18 uses). The step ladders feature was the most regularly used, with most adolescents using it 9 to 10 times during group treatment.

The average satisfaction score among adolescents was 11.3 (SD 1.4). Adolescents rated the step ladder (median rating of 7) and relaxation (median rating of 7) features of MindClimb as slightly more helpful in completing weekly exposure activities than the thinking traps feature (median rating of 6) (Table 4). The average satisfaction score among therapists was 10.0 (SD 2.0). Therapists reported slightly higher overall satisfaction with the app compared with adolescents (Table 4).

**Table 4:** Adolescent and therapist satisfaction with MindClimb.

Question			Responses, n (%)
**Adolescents**			
	**Q1. Satisfaction with the amount of help they received from** **MindClimb**		
		Quite dissatisfied	0 (0)
		Indifferent or mildly dissatisfied	0 (0)
		Mostly satisfied	6 (75)
		Very satisfied	2 (25)
	**Q2. Satisfaction with the kind of support they wanted from** **MindClimb**		
		No, definitely not	0 (0)
		No, not really	1 (13)
		Yes, generally	6 (75)
		Yes, definitely	1 (13)
	**Q3. Whether they would recommend** **MindClimb** **to a friend if they needed similar help**		
		No, definitely not	0 (0)
		No, I don’t think so	0 (0)
		Yes, I think so	5 (63)
		Yes, definitely	3 (38)
	**Q4. Satisfaction with the amount of support they received from** **MindClimb** **to** **help them deal more effectively with their anxiety**		
		No, it seemed to make things worse	0 (0)
		No, it didn’t really help	0 (0)
		Yes, it helped somewhat	5 (63)
		Yes, it helped a great deal	3 (38)
	**Q5. Whether they would use** **MindClimb** **again if they were to seek help again**		
		No, definitely not	0 (0)
		No, I don’t think so	0 (0)
		Yes, I think so	6 (75)
		Yes, definitely	2 (25)
**Therapists**			
	**Q1. Satisfaction with the amount of contributions** **MindClimb** **made to each treatment**		
		Quite dissatisfied	1 (33)
		Indifferent or mildly dissatisfied	0 (0)
		Mostly satisfied	1 (33)
		Very satisfied	1 (33)
	**Q2. Whether they would recommend** **MindClimb** **to a colleague who was looking to use an app in their clinical practice**		
		No, definitely not	0 (0)
		No, I don’t think so	0 (0)
		Yes, I think so	0 (0)
		Yes, definitely	3 (100)
	**Q3. Whether they would use** **MindClimb** **in the future with other patients**		
		No, definitely not	0 (0)
		No, I don’t think so	0 (0)
		Yes, I think so	1 (33)
		Yes, definitely	2 (67)
	**Q4. General satisfaction with using** **MindClimb** **as part of their clinical practice**		
		Quite dissatisfied	0 (0)
		Indifferent or mildly dissatisfied	0 (0)
		Mostly satisfied	1 (33)
		Very satisfied	2 (67)

MindClimb experience interviews with adolescents identified that they all participated in the study to see if the app could help them manage their anxiety. All the adolescents felt confident in their ability to use the app, found it easy to use, and stated that experience was not needed to use it. One adolescent noted that some prior CBT sessions would be required so that treatment terminology and skills (eg, step ladders) were learned prior to MindClimb use. A selection of adolescent quotes on the MindClimb app, grouped by our a priori categories (experiences, barriers, facilitators), are provided in Textbox 1. All the adolescents felt that the app fit well with their usual approach to treatment activities and was useful to helping complete treatment activities outside of group sessions. Moreover, 6 of the adolescents reported that they did not experience any negative outcomes associated with using MindClimb. All 8 adolescents found the reminders for use (eg, “miss you” and “where have you been?”) that were built into the app were helpful for use and necessary to ensure that they used the app regularly. Six adolescents found that they would forget to use the app if they had a busy day, if they “weren’t in the right head space—like feeling mad,” or if they had a lot of school homework.

**Adolescent perspectives on using the MindClimb app.**

**Experiences with MindClimb during treatment**

- “I used it for a Step Ladder on how to be comfortable around new people. One of the steps was to sit beside someone on the bus. I felt accomplished when I did it.” [Participant 2]
- “I used it when starting a new dance class. In the car I used the relaxation sounds/techniques.” [Participant 5]
- “It is nice that it is on my phone and I can use it whenever I need it. It is easy and helpful.” [Participant 3]
- “Helped a lot. Not needing to look at book [group materials]. Have it with me all the time on the phone.” [Participant 4]
- “Pretty important. Step Ladders in group had reminders and would help me remember to do therapy homework which was helpful.” [Participant 6]

**Challenges and barriers to use**

- “Sometimes worried if making stepladder wrong. Put two activities in same stepladder but figured it out.” [Participant 6]
- “Low to mid was more helpful in terms of anxiety level using app. [When] very worried/nervous, [the app] did not help as anxiety too high.” [Participant 8]
- “It can get boring with only Step Ladders. Being able to get different coloured characters and new accessories [rewards] would make me use it more.” [Participant 2]

**Facilitators for use**

- “Reminders were good. Step Ladders helped. Homework [from the group] is stressful as is, [the app] helped remind to do it.” [Participant 8]
- “Relaxed, planned out [prepared], organized.” [Participant 7]
- “Reminded me of the things I learned in group. What they teach is there [in the app] all the time.” [Participant 4]
- “… it was really easy to open up app anywhere and use it.” [Participant 1]
- “Happy when got points and new [characters].” [Participant 6]

MindClimb experience interviews with therapists identified that they were interested in integrating technology into group sessions. One of the therapists described:

We think it would help in engaging in using skills. Also, the importance of having your skills and tools with you at all times, and youth always have their phones with them to refer to. In future, we should make it more part of the group and have youth keep their phones out to use during the group sessions.

Therapist 3

Two of the therapists noted that the views of their patients were important to app use:

If they didn’t find it useful then I would be less likely to try again in the future.

Therapist 2

The 3 therapists described feeling confident in incorporating the app into treatment sessions, stating that the app was easy to use and intuitive. However, reminding patients to use it in sessions, including detailing app use in the treatment manuals and having therapists model use during group sessions, were described as approaches for ensuring routine use and maximizing app contributions, and 1 therapist noted that not having access to their patients’ step ladders was challenging, as they liked to have copies on file (“ability to review/record information” [Therapist 1]). Selected quotes from the therapists’ experiences with MindClimb use are presented in Textbox 2, as are quotes on perceived benefits and limitations to use.

**Therapist perspectives on using the MindClimb app with adolescents during group cognitive behavioral therapy (CBT).**

**Experience using MindClimb**

- “So in group setting [I] was a little hesitant to ask and have them use it. Did not want to make them feel uncomfortable. Worried that would get distracted with navigating app instead of content. Would be great to have the app on computer or iPad on smart board—project on screen and follow on the large screen. This would be easier to help visually.” [Therapist 1]
- “Sometimes forgot to remind people to use it. Need reminders for clinicians and possibly integrate in manual so that you remember to incorporate in each part of CBT.” [Therapist 3]
- “No ethical or legal issues beyond what youth already record in their book or notes, need to keep it confidential and not able to [be] accessed by others. In most cases book/paper is less confidential as lying around.” [Therapist 3]

**Benefits to using MindClimb during treatment**

- “Much more helpful for youth to use their skills in the moment- huge plus. Also most teens are digital oriented with their phone, so more likely to use than paper, and more fun. More [discreet] as well… Most teens will forget workbook but very few forget their phones.” [Therapist 1]
- “Main benefits are that the material and skills are always accessible and anxiety can happen anywhere. Also great for reminding youth to practice. [Helps] the clinician in having skills and tools more readily available for practice (like not forgetting their homework sheet).” [Therapist 3]

**Limitations to using MindClimb during treatment**

- “Only disadvantage is not being able to share stepladders… Realistic thinking was [also] a challenge as the app did not fit as well with this skill.” [Therapist 1]
- “I think having them put in the reminders in-session would have been better; to walk them through how to use it with their own examples. Again, easier done in an individual than a group setting.” [Therapist 2]
- “[Need] preplanning with group to incorporate app into group structure. More info structure on how to do it in the group as to how they would use it. Also need to make sure they are using the app for group and not something else on their phone.” [Therapist 1]
- “More screen time.” [Therapist 3]

## Discussion

### Principal Results

In this study, a multiphasic user-centered design approach allowed our team to develop, design, and test the MindClimb app with input from designers, developers, adolescents, and therapists using multiple approaches. Guiding principles for app development were the promotion of autonomy and ownership of treatment goals outside of formal treatment sessions, the focus on real-time skills practice, the personalization of treatment, and the simplicity of use under stress. The design of the app incorporated core components in a successful digital mental health tool that was secure and co-designed by stakeholders (adolescent users and clinicians) and that integrated evidence-based skills and usability [28]. The app was designed with high rigor in 3 stages of development and had high usability ratings by users (adolescents) and app developers, with only minor errors that were corrected before the final version. In our real-world usability evaluation, we found that adolescents and therapists were satisfied with the MindClimb app and found it easy to use and helpful in practicing CBT skills during treatment. Adolescents found the relaxation strategies and the step ladders with reminders to practice to be the most helpful components of the app. Therapists identified the benefits of having this app to help youth practice skills between CBT sessions and encouraged further integration of app usage into CBT sessions to encourage regular app usage. Both therapists and adolescents found the tools on the app easy to use and the most relevant to CBT practice. Step ladders and relaxation strategies were the two most used components of the app, and adolescents reported using the app between CBT sessions.

### Limitations

MindClimb was designed to be simple and easy to use between CBT sessions to plan and complete real-time exposure activities using skills (cognitive, relaxation, exposure practice, and reward) learned in treatment. As it was not intended to be a broad-scope, stand-alone treatment app, it includes some but not all of the recommended evidence-based treatment components for anxiety disorders: psychoeducation, self-monitoring, cognitive skills, problem solving, exposure activities, and contingency management. In a recent review of the content in smartphone apps marketed for child and adolescent anxiety, Bry et al [29] concluded that there are few comprehensive, evidence-based self-management apps available for use. We propose that fit for purpose also needs to be considered alongside app content to ensure that the proposed intent of an app is supported by its content and that evaluations of an app are also consistent with its intent. In this regard, we feel that MindClimb has been designed for a specific purpose for use by adolescents (users) in treatment and CBT therapists, and its functions reflect this purpose.

The results of MindClimb implementation are derived from a small sample of adolescents and therapists and should be interpreted with this limitation in mind. We were unable to recruit our intended sample size for phase 3 of the project, which would have allowed us to study app use over a more diverse user population (including, potentially, male users). Recruitment was challenging, as there were not as many CBT groups offered for anxiety during the study period due to a decrease CBT clinician availability. Future implementation studies of MindClimb (and other mHealth apps) need to examine the penetrance of MindClimb in clinical practice and include more diverse populations of users (reflected in gender identity, type of anxiety disorder and treatment setting, etc).

### Comparison With Prior Work

A recent review by Wozney and colleagues [30] indicated that acceptability is the most commonly investigated implementation outcome among studies of e-mental health care technology for adolescent depression and anxiety. The development and evaluation of MindClimb contributes additional methods and data on app learnability and initial usability. The field of e-mental health care technology will continue to benefit from studying a broader range of implementation outcomes. For apps such as MindClimb that are intended for use alongside therapy, important implementation outcomes include fidelity (the degree to which an app was implemented during CBT as intended), penetration (the extent of app use within a treatment setting), and sustainability (the extent to which an app’s use was maintained in a treatment setting) [31]. Feedback from therapists in this study suggested that workflow and integration issues are critical for embedding new technology in clinical care. Like our study, in other mHealth studies, technology has been regarded as useful, easy, and relevant to CBT practice, but support during use, other commitments, perceived relevance, and difficulty of use can be factors in whether an adolescent uses and continues to use a technology [32,33]. Implementation studies examining fidelity, penetration, and sustainability can introduce new understanding of the impact of apps on the course of treatment and on therapist roles, responsibilities, and workflow and inform the development of tailored implementation strategies to support app use during therapy. Such evaluations can occur within studies testing the impact of the technology on adolescent health outcomes. This hybrid approach to studying intervention effectiveness and implementation [34] offers an efficient and comprehensive approach to studying mHealth technologies in real-world clinical settings.

### Conclusions

MindClimb was developed and tested by adolescent users of CBT and therapists in cooperation with app developers. This approach resulted in an mHealth app that was relevant, accepted, and used by adolescents during CBT for anxiety. Evaluation of the use of this app in a clinical practice setting demonstrated that adolescents and therapists generally felt it was helpful and easy to use for CBT practice outside of therapy sessions. As exposure practice is the core component to anxiety CBT treatment and often reported as the most challenging to practice, the app shows promise in helping encourage adolescents to use these skills outside of therapy sessions. Having CBT skills and tools at all times on their phones provides adolescents the opportunity to practice skills more often between sessions. MindClimb increased access to evidence-based CBT skills outside of the formal therapy sessions in a format accepted and used by teenagers. Clinicians found the app helpful in encouraging practice but sometimes forgot to remind their patients; they recommended integrating app usage into the CBT treatment manual. Implementation studies with larger youth samples that examine the integration of technology in clinical care and the impact of the app plus CBT on clinical care processes and patient outcomes are now necessary.

## Appendix

Multimedia Appendix 1 Questions asked during initial consultations to develop the MindClimb app.

Multimedia Appendix 2 Questions asked during the Think Aloud activity.

Multimedia Appendix 3 MindClimb usage survey.

Multimedia Appendix 4 Satisfaction questions.

Multimedia Appendix 5 User experience interview questions for therapists.

Multimedia Appendix 6 User experience interview questions for adolescents.

## Abbreviations

CBT: cognitive behavioral therapy
EMI: ecological momentary intervention
mHealth: mobile health
NYAC: National Youth Advisory Committee
PIN: personal identification number
SCARED: Screen for Child Anxiety Related Emotional Disorders
SRS: severity ranking scale
SUS: System Usability Scale
TDF: Theoretical Domains Framework
