# Supplementary material for: A Mobile Phone–Based App for Use During Cognitive Behavioral Therapy for Adolescents With Anxiety (MindClimb): User-Centered Design and Usability Study
Source: JMIR Mhealth Uhealth. 2020 Dec 8;8(12):e18439. doi: 10.2196/18439 (PMC7755529; doi:10.2196/18439)
Supplement: Multimedia Appendix 1 [file mhealth_v8i12e18439_app1.docx]

Multimedia Appendix 1. Questions asked during initial consultations to develop the *MindClimb* app.

| Section 1: Questions posed during consultation for the pre-design prototype |
| --- |
| 1. What capabilities should the app have? |
| 2. Overall, how should the app ‘look and feel’? |
| 3. What kinds of colors and fonts should the app have? |
| 4. What should the experience with the app be like? |
| Section 2: Questions posed during consultation for the high-fidelity prototype |
| 1. Do you understand what the app is for? (Yes/No) |
| 2. How hard was it to create an event? (scale of 1-5 with 5 being hard and 1 being easy) |
| 3. How easy was it to navigate through the app? (scale of 1-5 with 5 being hard and 1 being easy) |
| 4. Which terms to you prefer?  a) Fear ladder, Step ladder or Other?  b) Thinking traps, Anxious thinking or Other?  c) Deep breathing, Belly breathing or Other? |
| 5. Which color scheme do you prefer? |
| 6. Are there photos or images that you liked or didn’t like? |
| 7. Are there things you found confusing? |
| 8. Are there things you would have done differently? |
| 9. Any other comments? |
