# Supplementary material for: A Mobile Phone–Based App for Use During Cognitive Behavioral Therapy for Adolescents With Anxiety (MindClimb): User-Centered Design and Usability Study
Source: JMIR Mhealth Uhealth. 2020 Dec 8;8(12):e18439. doi: 10.2196/18439 (PMC7755529; doi:10.2196/18439)
Supplement: Multimedia Appendix 2 [file mhealth_v8i12e18439_app2.docx]

Multimedia Appendix 2. Questions asked during the Think Aloud activity.

| Cycle 1 |
| --- |
| 1. How successful were you in creating your ladder? |
| 2. How hard did you have to work to create a ladder? (provide examples) |
| 3. In what ways did the app make it easy to create the ladder? |
| 4. In what ways did the app make it hard to complete the ladder? |
| 5. If you waited 3 weeks before using this app again, do you think you would remember how to use it to create a ladder? |
| 6. How often do you think you would use this app in TAG (Treatment of Anxiety Group)? |
| 7. How often do you think you would use this app on your own? |
| 8. What would help you use this app more often? Remember the way to input information? |
| 9. Were there any problems with app installation? |
| 10. Did you experience any errors, mistakes, difficulty locating things in the app as you tried to create a ladder? |
| 11. Do you have any additional comments/suggestions? |
| Cycle 2 |
| 1. How did you use the app since the last time we met? |
| 2. How long did it take you to learn how to use the app? |
| 3. What information or tools within the app helped you the most? least? |
| 4. What app features would have helped you be more successful (e.g., faster, less steps, use the app over and over again) |
| 5. How rewarding or enjoyable was the app to use? |
| 6. Describe any features of the app you would change. Why? |
| 7. How relevant was the content of the app for being in TAG? |
| 8. Were there any features you wanted to customize in the application but were not able to? How did that affect your overall satisfaction? |
| 9. Do you have any additional comments/suggestions? |
