# Supplementary material for: A Mobile Phone–Based App for Use During Cognitive Behavioral Therapy for Adolescents With Anxiety (MindClimb): User-Centered Design and Usability Study
Source: JMIR Mhealth Uhealth. 2020 Dec 8;8(12):e18439. doi: 10.2196/18439 (PMC7755529; doi:10.2196/18439)
Supplement: Multimedia Appendix 3 [file mhealth_v8i12e18439_app3.docx]

Multimedia Appendix 3. *MindClimb* usage survey.

This survey is used to assess how often a patient has used *MindClimb* since their previous therapy session, as well as which features they used. Clinicians should complete this survey with their patient at every session.

Youth Name: ___________________________ Date Completed: ______________

Clinician Name: _________________________ Treatment session #: __________

Please ask your patient the following questions:

1. How many times did you use the Step Ladder feature of *MindClimb* this week? (circle one)

| 0 | 1 | 2 | 3 | 4 | 5+ |
| --- | --- | --- | --- | --- | --- |

2. On scale of 1 to 10, how well did the Step Ladder help you do your exposure? (circle one)

| 1 | 2 | 3 | 4 | 5 | 6 | 7 | 8 | 9 | 10 |
| --- | --- | --- | --- | --- | --- | --- | --- | --- | --- |

3. How many times did you use the Relaxation feature of *MindClimb* this week? (circle one)

| 0 | 1 | 2 | 3 | 4 | 5+ |
| --- | --- | --- | --- | --- | --- |

4. On a scale of 1 to 10, how well did the Relaxation feature help you? (circle one)

| 1 | 2 | 3 | 4 | 5 | 6 | 7 | 8 | 9 | 10 |
| --- | --- | --- | --- | --- | --- | --- | --- | --- | --- |

5. How often did you use the Thinking Traps feature of *MindClimb* this week? (circle one)

| 0 | 1 | 2 | 3 | 4 | 5+ |
| --- | --- | --- | --- | --- | --- |

6. On a scale of 1 to 10, how well did the Thinking Traps feature help you? (circle one)

| 1 | 2 | 3 | 4 | 5 | 6 | 7 | 8 | 9 | 10 |
| --- | --- | --- | --- | --- | --- | --- | --- | --- | --- |
|  |  |  |  |  |  |  |  |  |  |
