# Supplementary material for: A Mobile Phone–Based App for Use During Cognitive Behavioral Therapy for Adolescents With Anxiety (MindClimb): User-Centered Design and Usability Study
Source: JMIR Mhealth Uhealth. 2020 Dec 8;8(12):e18439. doi: 10.2196/18439 (PMC7755529; doi:10.2196/18439)
Supplement: Multimedia Appendix 4 [file mhealth_v8i12e18439_app4.docx]

Multimedia Appendix 4. Satisfaction questions.

Adolescent Satisfaction Questionnaire

Name: Date:

This survey is used to determine how satisfied you are with *MindClimb*. Each person participating in the study should complete this questionnaire after using *MindClimb* for approximately 7 CBT sessions. We are interested in your honest opinions, whether they are positive or negative. Please answer all of the questions.

1. How satisfied are you with the amount of help you received from *MindClimb*?

___ Quite dissatisfied

___ Indifferent or mildly dissatisfied

___ Mostly satisfied

___ Very satisfied

2. Did you get the kind of support you wanted from *MindClimb*?

___ No, definitely not

___ No, not really

___ Yes, generally

___ Yes, definitely

3. If a friend were in need of similar help, would you recommend *MindClimb* to him or her?

___ No, definitely not

___ No, I don’t think so

___ Yes, I think so

___ Yes, definitely

4. Has the support you received from *MindClimb* helped you to deal more effectively with your anxiety?

___ Yes, it helped a great deal

___ Yes, it helped somewhat

___ No, it really didn’t help

___ No, it seemed to make things worse

5. If you were to seek help again, would you use *MindClimb*?

___ No, definitely not

___ No, I don’t think so

___ Yes, I think so

___ Yes, definitely

Please give your completed questionnaire to your therapist. *THANK YOU!*

Therapist Satisfaction Questionnaire

Name: Date:

This survey is used to determine how satisfied you are with *MindClimb* in your clinical practice. Each clinician participating in the study should complete this questionnaire after using *MindClimb* for approximately 7 CBT sessions. We are interested in your honest opinions, whether they are positive or negative. Please answer all of the questions.

1. How satisfied are you with the amount of contributions *MindClimb* made to each treatment session?

___ Quite dissatisfied

___ Indifferent or mildly dissatisfied

___ Mostly satisfied

___ Very satisfied

2. If a colleague were looking to use an app in their clinical practice, would you recommend *MindClimb* to him or her?

___ No, definitely not

___ No, I don’t think so

___ Yes, I think so

___ Yes, definitely

3. Would you use *MindClimb* in the future with other patients?

___ No, definitely not

___ No, I don’t think so

___ Yes, I think so

___ Yes, definitely

4. In an overall, general sense how satisfied are you with using *MindClimb* as a part of your clinical practice?

___ Very satisfied

___ Mostly satisfied

___ Indifferent or mildly dissatisfied

___ Quite dissatisfied

Place completed questionnaire in drop-box located in staff room. *THANK YOU!*
