# Supplementary material for: A Mobile Phone–Based App for Use During Cognitive Behavioral Therapy for Adolescents With Anxiety (MindClimb): User-Centered Design and Usability Study
Source: JMIR Mhealth Uhealth. 2020 Dec 8;8(12):e18439. doi: 10.2196/18439 (PMC7755529; doi:10.2196/18439)
Supplement: Multimedia Appendix 5 [file mhealth_v8i12e18439_app5.docx]

Multimedia Appendix 5. User experience interview questions for therapists.

Knowledge

1. Did your patient use *MindClimb* in between treatment sessions?

2. Why did you choose to use *MindClimb* with your patient? (If they did not use: is there a reason why you did not use *MindClimb*?)

Skills

1. Can you describe a typical situation when your patient used *MindClimb*? Did not use *MindClimb*, and describe why?

2. Now that your patient has tried MindClimb, what skills are needed to use *MindClimb* as part of CBT?

3. How much experience/expertise do you think one needs to use *MindClimb* as part of CBT?

Social/professional role and identity, and social influences

1. How might views/opinions of colleagues, patients, and professional groups influence your decision to use *MindClimb* as part of CBT?

2. Now that your patient has tried *MindClimb*, describe any legal or ethical issues in its use.

Beliefs about capabilities

1. How confident did you feel in your ability to use *MindClimb* with your patient as part of CBT?

2. What was your experience using *MindClimb* (Consider the setting (using at home/school, using in individual treatment session, using during group session: Were there specific problems with the application? Were there specific advantages?)

3. What would help your patient use the *MindClimb* as part of CBT?

Beliefs about consequences

1. What do you think are the consequences of using *MindClimb* as part of CBT? (What do you think the benefits of using *MindClimb* are (i.e., patient benefits, parent benefits, clinician benefits, administrative benefits, other?)

2. Now that your patient has tried *MindClimb*, is there any harm that can occur or disadvantage as a result of using *MindClimb* as part of CBT?

3. Do the potential benefits outweigh the potential harms for using *MindClimb* as part of CBT?

4. Is there any incentive/disincentive that you can think of that influence whether or not a patient/clinician would use MindClimb as part of CBT?

Motivation and goals

1. How important is *MindClimb* for helping your patient complete CBT activities outside of treatment sessions?

2. Is *MindClimb* compatible with your usual approach to practicing activities/treatment? Why/why not?

Memory, attention and decision process

1. What guided your decision to use *MindClimb* as part of CBT? What went through your mind?

2. Was it easy or difficult to remember to use *MindClimb* between treatment sessions? What would help make it easy to remember to use it?

3. Were there situations when it was difficult to use *MindClimb*? Can you describe what it was about those situations that made it difficult?

4. Did you sometimes forget to use *MindClimb*? When did you forget?

Environmental context and resources

1. Were there competing tasks or time constraints that influenced your decision to use *MindClimb*?

Emotion

1. Did using *MindClimb* result in an emotional response in you? (Did you ever feel worried or concerned about using it? Did these worries affect your decision to use it?)

Behavioural regulation and nature of the behavior

1. What do you think is needed to ensure that you regularly use MindClimb as part of CBT? (specific to you, your health centre)

2. For new patients starting with CBT for anxiety, how would you approach CBT now that you have used *MindClimb*?
