# Supplementary material for: A Mobile Phone–Based App for Use During Cognitive Behavioral Therapy for Adolescents With Anxiety (MindClimb): User-Centered Design and Usability Study
Source: JMIR Mhealth Uhealth. 2020 Dec 8;8(12):e18439. doi: 10.2196/18439 (PMC7755529; doi:10.2196/18439)
Supplement: Multimedia Appendix 6 [file mhealth_v8i12e18439_app6.docx]

Multimedia Appendix 6. User experience interview questions for adolescents.

1. Did you use *MindClimb* in between treatment sessions?

Yes [go to question 2, and 4-17]

No [go to question 3 only and end interview]

2. Why did you choose to use *MindClimb*?

3. Why did you not use *MindClimb*?

4. Please describe a time when you used *MindClimb*. Can you tell us why you used it?

5. How much experience or expertise do you think someone needs to use *MindClimb*?

6. How confident did you feel in your ability to use *MindClimb*?

7. Is there anything that would help you use *MindClimb* as part of your treatment?

8. What are the benefits of using *MindClimb* during treatment?

9. What are the negative outcomes that can occur by using *MindClimb*?

10. In your opinion, do the benefits of using *MindClimb* outweigh the negatives?

11. How is important was *MindClimb* for helping you complete treatment activities outside of sessions with your therapist?

12. Did *MindClimb* fit well with your usual approach to practicing your treatment activities? Why or why not?

13. Was it easy or difficult to remember to use *MindClimb* between CBT sessions? What would help make it easy to remember to use it?

14. Were there situations when it was difficult to use *MindClimb*? Can you describe what it was about those situations that made it difficult?

15. Did you sometimes forget to use *MindClimb*? When did you forget?

16. Were there any common feelings that appeared whenever you used *MindClimb*? Did you ever feel worried or concerned about using it? If, so did these worries affect your decision to use it?

17. What do you think is needed to ensure that you regularly use *MindClimb* as part of your treatment?
